# Fetal sexual dimorphism of maternal thyroid function parameters during pregnancy, a single center retrospective real-world study

**DOI:** 10.3389/fendo.2024.1431621

**Published:** 2024-08-16

**Authors:** Meiqin Wu, Chunping Hu, Dan Huang, Hao Ying, Jing Hua

**Affiliations:** ^1^ Shanghai Key Laboratory of Maternal Fetal Medicine, Department of Women’s and Children’s Health Care, Shanghai First Maternity and Infant Hospital, School of Medicine, Tongji University, Shanghai, China; ^2^ Department of Spine Surgery, Honghui Hospital, Xi’an Jiaotong University, Xi’an, Shaanxi, China; ^3^ Shaanxi Key Laboratory of Spine Bionic Treatment, Xi’an, Shaanxi, China; ^4^ Medical College, Soochow University, Suzhou, Jiangsu, China; ^5^ Department of Obstetrics, Shanghai First Maternity and Infant Hospital, School of Medicine, Tongji University, Shanghai, China

**Keywords:** fetal sex, thyroid function, pregnancy, reference intervals, real-world study

## Abstract

**Introduction:**

Thyroid function during pregnancy fluctuates with gestational weeks, seasons and other factors. However, it is currently unknown whether there is a fetal sex-specific thyroid function in pregnant women. The purpose of this study was to investigate the fetal sex differences of maternal thyroid-stimulating hormone (TSH) and free thyroxine (FT4) in pregnant women.

**Methods:**

This single-center retrospective real-world study was performed by reviewing the medical records of pregnant women who received regular antenatal health care and delivered liveborn infants in Shanghai First Maternity and Infant Hospital (Pudong branch), from Aug. 18, 2013 to Jul. 18, 2020. Quantile regression was used to evaluate the relationship between various variables and TSH and FT4 concentrations. The quantile regression also evaluated the sex impact of different gestational weeks on the median of TSH and FT4.

**Results:**

A total of 69,243 pregnant women with a mean age of 30.36 years were included. 36197 (52.28%) deliveries were boys. In the three different trimesters, the median levels (interquartile range) of TSH were 1.18 (0.66, 1.82) mIU/L and 1.39 (0.85, 2.05) mIU/L, 1.70 (1.19, 2.40) mIU/L; and the median levels (interquartile range) of FT4 were 16.63 (15.16, 18.31) pmol/L, 14.09 (12.30, 16.20) pmol/L and 13.40 (11.52, 14.71) pmol/L, respectively. The maternal TSH upper limit of reference ranges was decreased more in mothers with female fetuses during gestational weeks 7 to 12, while their FT4 upper limit of the reference ranges was increased more than those with male fetuses. After model adjustment, the median TSH level was 0.11 mIU/L lower (*P* <0.001), and FT4 level was 0.14 pmol/L higher (*P* <0.001) for mothers with female fetuses than those with male fetuses during gestational weeks 9 to 12.

**Discussion:**

We identified sexual dimorphism in maternal thyroid function parameters, especially during 9-12 weeks of pregnancy. Based on previous research, we speculated that it may be related to the higher HCG levels of mothers who were pregnant with girls during this period. However, longitudinal studies are needed to determine if fetal sex differences impact the maternal thyroid function across pregnancy.

## Introduction

Changes in the maternal endocrine environment during pregnancy are complex, involving products from multiple sources such as mother, fetus, and placenta. Thyroid physiology undergoes major changes during pregnancy, including the increase in thyroxine binding globulin, and the degradation of thyroid hormone by placental type 3 deiodinase (D3), and the stimulation of thyroid gland by high concentrations of human chorionic gonadotropin (hCG) (1, 2). These important changes during pregnancy are necessary for a successful pregnancy and a healthy baby. However, the general physiological changes in thyroid hormone levels make it difficult to distinguish between normal and abnormal hormonal values (3). In addition, the definitions of both overt and subclinical thyroid dysfunction have changed considerably over the past few years in different versions of the guidelines (4–6). Therefore, accurate assessment of thyroid function during pregnancy is challenging (7, 8).

Many of the previous studies have focused on the impact of maternal thyroid function during pregnancy on the outcome of pregnancy and the thyroid level of offspring. In turn, Fu et al. ‘s research showed that thyroid hormone levels and central sensitivity to thyroid hormones were influenced by age and seasonal fluctuations among women of reproductive age (9). Also, TSH secretion exhibits a clear daily time-dependent variations (10). There is also increasing evidence indicating that the sex of the fetus may alter maternal metabolic milieu, such as the maternal glucose metabolism during pregnancy (11, 12). But there is scant information regarding the impact of fetal sex differences on maternal thyroid function. Chen et al. ‘s research showed that there were no significant differences in maternal serum or cord blood thyroid hormone levels between male and female births (13). In a Belgian study, women pregnant with a male fetus have been reported to have slightly but significantly higher thyroid stimulating hormone (TSH) levels (14).

With the contradictory findings, we conducted a single center retrospective real-world study in reproductive-aged women in coastal city in east China. The purpose of this study was to further assess the effects of different fetal sex on maternal TSH and free thyroxine (FT4) during pregnancy base on a large sample. Our research may add benefit to the field around allowing better determination of a sex-specific thyroid function reference range.

## Materials and methods

### Design and data collection

**Figure 1:**
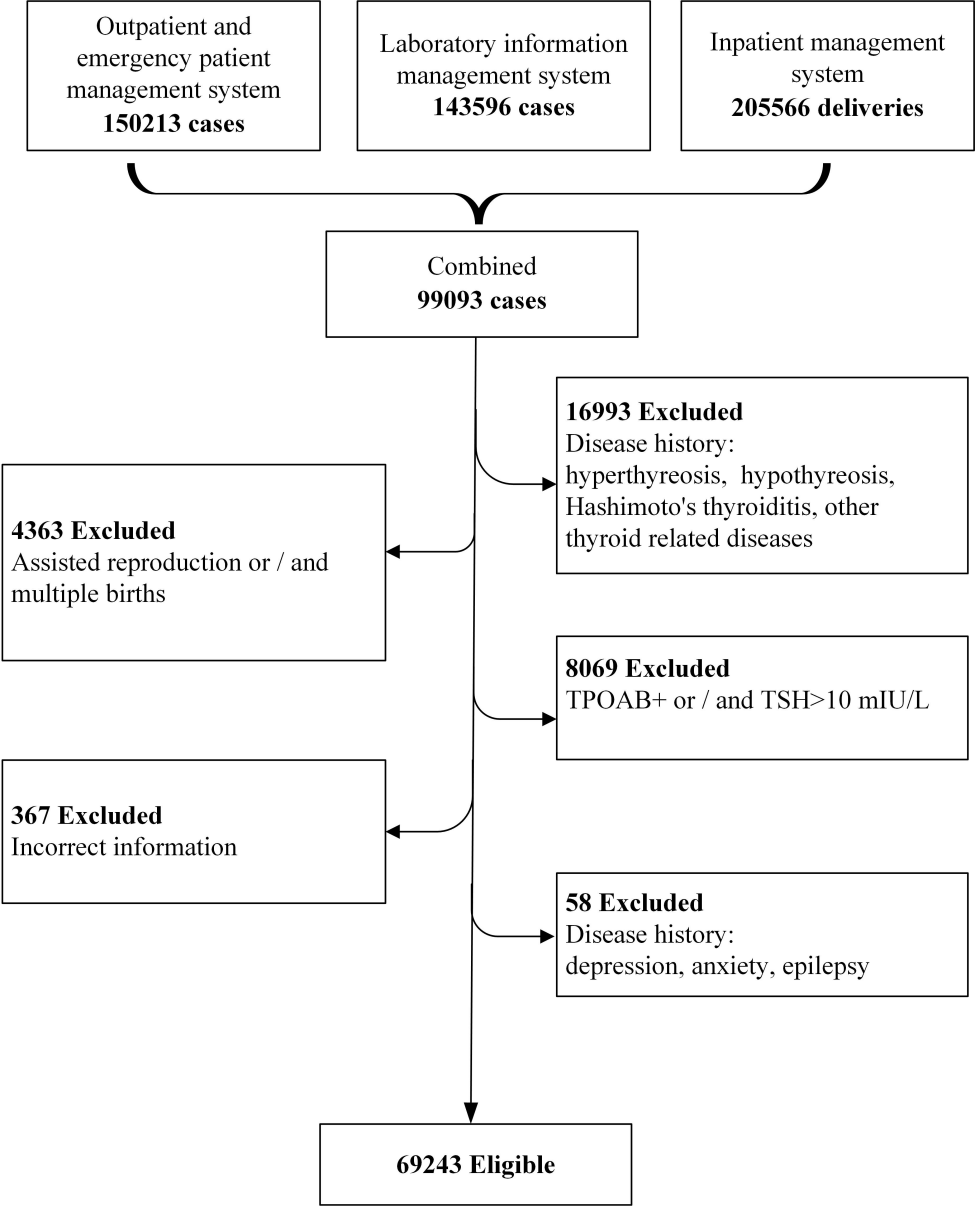
Flowchart of the study population.

This study was conducted in Shanghai, China, which has a subtropical monsoon climate, with four distinct seasons, and more than 27 million residents with about 136, 300 newborns in 2020 (15, 16). The study subjects were selected from a real-world clinical data, who delivered in Shanghai First Maternity and Infant Hospital (Pudong branch), Tongji University School of Medicine from Aug. 18, 2013 to Lul. 18, 2020. A total of 150,213 women were identified as potential participants. We excluded those without available detailed pregnancy check-up information and thyroid function biological measurements during pregnancy. In addition, the pregnant women with thyroid related diseases (hyperthyreosis, hypothyreosis, Hashimoto’s thyroiditis, elevated thyroid peroxidase antibodies (TPOAb)) and diseases that affect pregnancy outcome (depression, anxiety, and epilepsy) were all excluded. Finally, 69,243 cases were eligible for further analysis (**Figure 1**).

All the original data were obtained from the outpatient and emergency patient management system, laboratory information management system and inpatient management system. The data were combined by unique ID, anonymously processed and analyzed by a dedicated statistician. The study was approved by the Ethics Committee of Shanghai First Maternity and Infant Hospital (No. KS20172).

### Group classification and anthropometrics

Pregnant women would have more than one thyroid function test during the whole pregnancy. To reduce the impact of other factors on thyroid function of pregnant women, we used the test results from their first sample collection. According to the time of the first thyroid function testing, the subjects were categorized into 3 different groups by the gestational age (GA), namely first trimester (<13 weeks, T1), second trimester (≥13 and <28 weeks, T2), and third trimester (≥28 weeks, T3). GA was calculated based on the date of sample collection and the date of last menstrual period (LMP). The exact collection date and time of each specimen were recorded for each subject by the laboratory information system and was divided into three time intervals: ante meridiem: 6:00-12:00; post meridiem: 12:00-18:00; nighttime: 18:00-6:00 (shown in **Table 1**).

**Table 1:** General population characteristics.

Characteristics	Statistics, M (SD)/N (%)
**Age (year)**	30.36 ± 3.79
**Gestation age (week)**	11.98 ± 4.55
**BMI (pre-pregnancy)**	21.85 ± 2.96
Ethnicity	
Han	67318 (98.19)
Hui	255 (0.37)
Man	248 (0.36)
Menggu	114 (0.17)
Miao	69 (0.10)
Tujia	146 (0.21)
Zhuang	86 (0.13)
Other	325 (0.47)
Fetal sex	
Male	36197 (52.28)
Female	33046 (47.72)
Pregnancy stage	
First trimester	43629 (63.00)
Second trimester	24858 (35.90)
Third trimester	756 (1.10)
Time interval	
Ante meridiem	65886 (95.15)
Post meridiem	3338 (4.82)
Nighttime	19 (0.03)
Season	
Spring	18264 (26.38)
Summer	16836 (24.31)
Autumn	17515 (25.30)
Winter	16628 (24.01)
TSH, median (IQR), mIU/L	
First trimester	1.18 (0.66, 1.82)
Second trimester	1.39 (0.85, 2.05)
Third trimester	1.70 (1.19, 2.40)
FT4, median (IQR), pmol/L	
First trimester	16.63 (15.16, 18.31)
Second trimester	14.09 (12.30, 16.20)
Third trimester	13.40 (11.52, 14.71)

M (SD), Mean (Standard Deviation); BMI, Body Mass Index; IQR, Interquartile Range.

First trimester: 1-12week; Second trimester: 13-28week; Third trimester: >28week.

Ante meridiem: 6:00-12:00; Post meridiem: 12:00-18:00; Nighttime: 18:00-6:00.

Spring, March-May; Summer, June-August; Autumn, September-November; Winter, December-February.

### Hormone assays

Maternal venous blood samples were collected in the outpatient/emergency department, and the laboratory analysis were completed within 24 hours. Serum TSH, FT4 and TPOAb were measured by using ADVIA Centaur instruments and kits (Siemens, Munich, Germany). TPOAb levels ≥60 U/mL were considered as elevated (17).

### Data analysis

Discrete variables were presented as number (N, %). For normally distributed continuous variables, values were expressed as mean ± SD, and median with interquartile range (IQR) for skewed continuous variables.

The statistical methodology employed in this study involved quantile regression to assess the relationship between various variables and the concentrations of TSH and FT4. The analysis was conducted at 5 specific percentiles (P2.5, P25, P50, P75, and P97.5) to capture potential variations in the relationships across different points of the distribution (18). Quantile regression also assessed sex-specific effects on TSH and FT4 medians across gestational weeks including crude and adjusted models, with the latter incorporating maternal age, pre-pregnancy BMI, pregnancy stage, ethnicity, season, and time interval as comprehensive covariates. The analysis was performed utilizing the rq() function from the quantreg (version:5.95) R package. A value of *P*<0.05 was considered statistically significant.

## Results

### Descriptive data for characteristics of the participants

The mean age and gestation age (standard deviation) of the enrolled 69,243 pregnant women was 30.36 (3.79) years and 11.98 (4.55) weeks, respectively. 36197 (52.28%) deliveries were boys. Most of the blood specimens for serum TSH and FT4 were collected in the ante meridiem (95.15%) and T1 (63.00%), with relatively average seasonal distribution. The median (interquartile range) of TSH, FT4 was 1.18 (0.66, 1.82) mIU/L and 16.63 (15.16, 18.31) pmol/L in T1, 1.39 (0.85, 2.05) mIU/L and 14.09 (12.30, 16.20) pmol/L in T2, 1.70 (1.19, 2.40) mIU/L and 13.40 (11.52, 14.71) pmol/L in T3, respectively (**Table 1**). Information on general characteristics including maternal ethnic distribution, body mass index (BMI) (kg/m^2^) pre-pregnancy were shown in **Table 1**.

### Sex-specific reference interval change by gestational age

**Figure 2:**
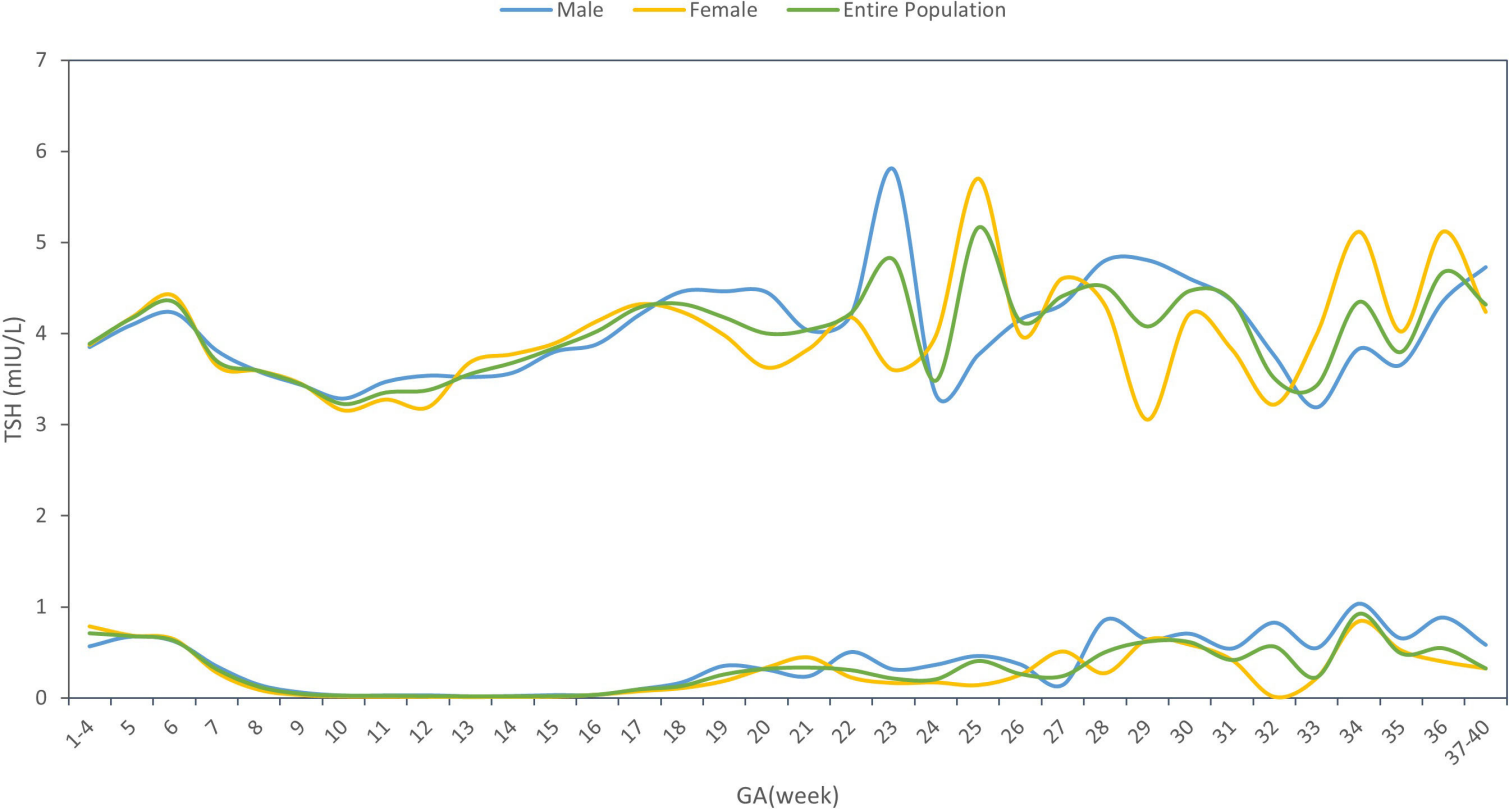
TSH reference interval by gestational age and sex.

**Figure 3:**
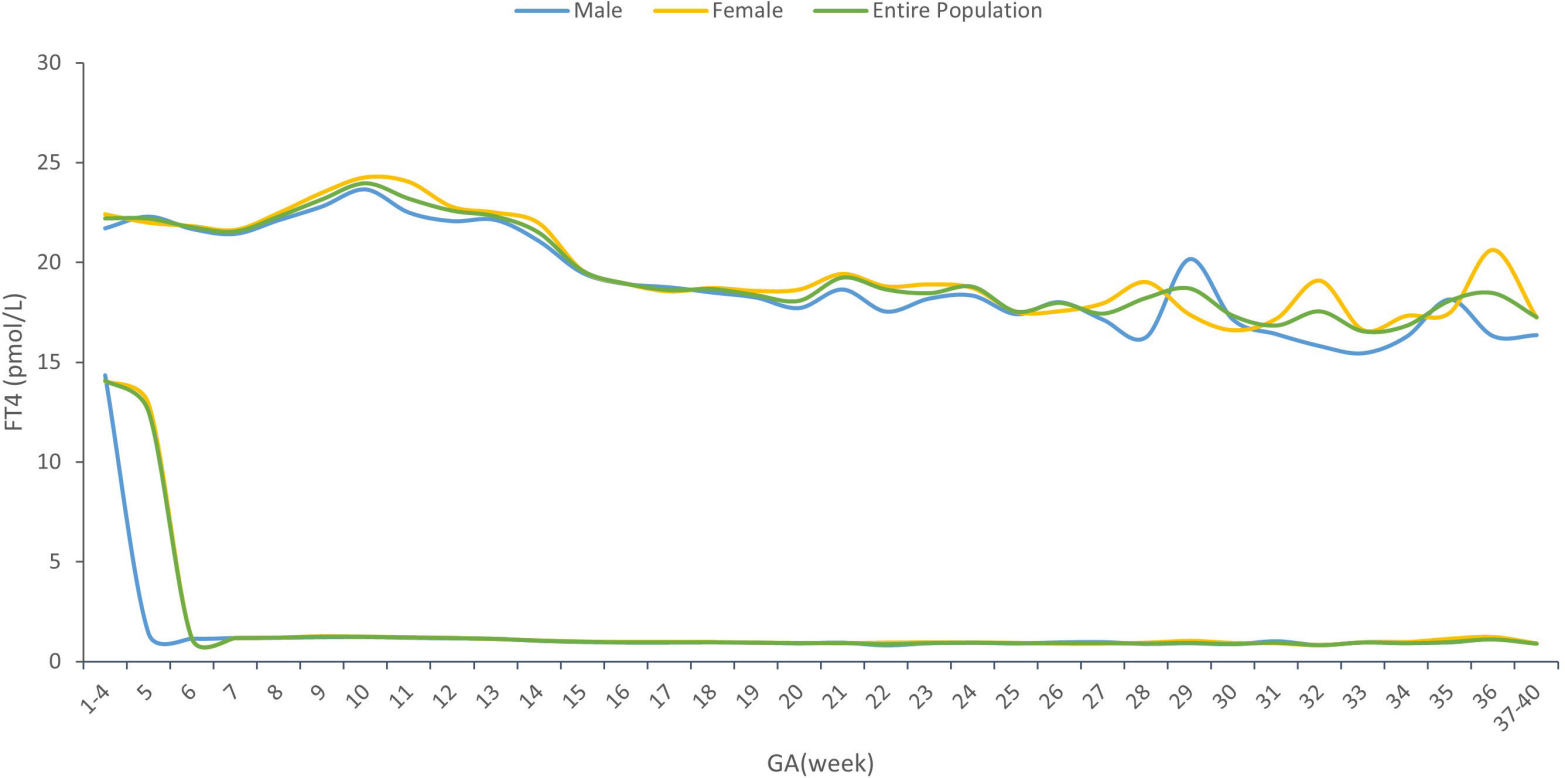
FT4 reference interval by gestational age and sex.

**Supplementary Material STable 1** showed the sex-specific sample size for each gestational week. The sex-specific TSH and FT4 95% reference interval ranges week by week throughout the prenatal stage were analyzed and displayed in **Table 2** and **Figures 2**, **3**, from which we can see that the TSH upper limit of reference ranges during gestational weeks 7 to 12 was decreased more in mothers with female fetuses, while at the same time, the FT4 upper limit of the reference ranges was increased more in mothers with female fetuses than those with male fetuses. At 13 gestation weeks there was a cross-over such that higher maternal TSH was observed in women bearing female fetuses beyond this time till 17 weeks of gestation. In the following gestational age, fluctuations of hormones in different sex groups were detected: mothers of male fetuses had elevated TSH upper limit of the reference ranges at 22 weeks of gestation, whereas mothers of female fetuses had elevations when assessed at 25, 34, and 36 weeks while their FT4 levels were generally less affected.

**Table 2:** Sex-specific reference interval of TSH and FT4 in the different gestational weeks.

GA* (week)	TSH (mIU/L)			FT4 (pmol/L)			
	Male (n)	Female (n)	Entire Population (n)	Male (n)	Female (n)	Entire Population (n)
1	0.48-3.67 (9)	1.48-2.61 (6)	0.49-3.60 (15)	15.43-21.58 (9)	16.44-19.36 (6)	15.45-21.49 (15)
2	2.39-2.39 (1)	1.15-4.59 (3)	1.11-4.58 (4)	16.50-16.50 (1)	16.75-20.29 (3)	16.51-20.23 (4)
3	0.83-2.75 (11)	1.11-2.63 (4)	0.85-2.75 (15)	14.18-22.22 (11)	15.37-21.10 (4)	14.29-22.13 (15)
4	0.76-3.84 (182)	0.80-4.18 (169)	0.77-3.96 (351)	13.67-21.61 (182)	13.46-22.29 (169)	13.57-21.96 (351)
5	0.64-4.09 (711)	0.68-4.33 (727)	0.65-4.28 (1438)	1.21-22.26 (711)	12.38-22.14 (727)	1.27-22.23 (1438)
6	0.50-4.02 (1381)	0.47-4.07 (1344)	0.48-4.06 (2725)	1.14-21.56 (1381)	1.14-21.63 (1344)	1.14-21.58 (2725)
7	0.26-3.72 (3161)	0.18-3.70 (2852)	0.23-3.72 (6013)	1.20-21.63 (3161)	1.16-22.01 (2852)	1.17-21.82 (6013)
8	0.10-3.56 (3586)	0.06-3.36 (3225)	0.08-3.49 (6811)	1.19-22.26 (3586)	1.22-22.89 (3225)	1.21-22.50 (6811)
9	0.04-3.33 (3512)	0.03-3.36 (3156)	0.03-3.34 (6668)	1.27-23.01 (3512)	1.30-24.00 (3156)	1.28-23.39 (6668)
10	0.02-3.38 (2944)	0.02-3.27 (2728)	0.02-3.31 (5672)	1.20-23.56 (2944)	1.22-24.61 (2728)	1.21-24.15 (5672)
11	0.03-3.60 (2530)	0.02-3.19 (2283)	0.02-3.38 (4813)	1.21-22.08 (2530)	1.19-23.68 (2283)	1.20-22.80 (4813)
12	0.03-3.49 (3122)	0.02-3.43 (2810)	0.02-3.47 (5932)	1.14-22.16 (3122)	1.18-22.44 (2810)	1.16-22.35 (5932)
13	0.02-3.49 (2720)	0.01-3.66 (2487)	0.02-3.54 (5207)	1.08-21.58 (2720)	1.09-22.39 (2487)	1.09-21.97 (5207)
14	0.02-3.68 (2269)	0.02-3.88 (2090)	0.02-3.81 (4359)	1.01-20.75 (2269)	1.02-20.63 (2090)	1.02-20.72 (4359)
15	0.03-3.87 (2648)	0.02-3.91 (2334)	0.02-3.87 (4982)	0.96-18.94 (2648)	0.99-19.20 (2334)	0.97-19.17 (4982)
16	0.05-4.13 (2271)	0.05-4.24 (2192)	0.05-4.20 (4463)	0.95-18.89 (2271)	0.97-18.76 (2192)	0.96-18.82 (4463)
17	0.11-4.22 (1741)	0.07-4.37 (1581)	0.10-4.26 (3322)	0.95-18.71 (1741)	0.97-18.59 (1581)	0.96-18.67 (3322)
18	0.22-4.55 (1370)	0.24-4.23 (1326)	0.22-4.35 (2696)	0.95-18.43 (1370)	0.95-18.96 (1326)	0.95-18.64 (2696)
19	0.34-4.42 (642)	0.18-3.89 (563)	0.26-4.07 (1205)	0.92-18.02 (642)	0.94-17.95 (563)	0.94-18.02 (1205)
20	0.25-4.03 (282)	0.42-3.66 (239)	0.34-3.88 (521)	0.94-18.08 (282)	0.92-18.12 (239)	0.92-18.08 (521)
21	0.55-4.18 (167)	0.26-4.23 (175)	0.29-4.22 (342)	0.84-17.80 (167)	0.91-19.18 (175)	0.87-18.76 (342)
22	0.31-3.97 (142)	0.18-4.12 (119)	0.20-4.09 (261)	0.85-17.27 (142)	0.97-19.00 (119)	0.93-18.86 (261)
23	0.40-4.92 (105)	0.45-3.82 (88)	0.42-4.00 (193)	0.90-18.55 (105)	0.96-16.84 (88)	0.92-18.48 (193)
24	0.41-3.50 (99)	0.17-4.69 (51)	0.24-4.01 (150)	0.96-17.07 (99)	0.95-19.50 (51)	0.96-17.41 (150)
25	0.51-4.44 (72)	0.10-5.88 (52)	0.31-5.74 (124)	0.91-18.05 (72)	0.93-17.55 (52)	0.91-18.02 (124)
26	0.55-4.16 (81)	0.33-3.71 (56)	0.49-4.00 (137)	0.96-17.00 (81)	0.88-17.64 (56)	0.92-17.40 (137)
27	0.09-5.24 (60)	0.36-4.83 (52)	0.22-5.09 (112)	0.89-16.84 (60)	0.90-17.90 (52)	0.89-17.42 (112)
28	0.69-4.14 (55)	0.40-3.50 (40)	0.49-4.14 (95)	0.95-19.50 (55)	1.01-19.28 (40)	0.95-20.15 (95)
29	0.63-4.73 (36)	0.36-3.91 (31)	0.50-4.47 (67)	0.91-17.07 (36)	1.05-17.44 (31)	0.93-17.44 (67)
30	0.53-4.45 (38)	0.58-4.41 (38)	0.54-4.50 (76)	0.90-16.99 (38)	0.85-20.06 (38)	0.87-17.39 (76)
32	0.64-3.51 (34)	0.02-3.21 (41)	0.06-3.43 (75)	0.94-16.10 (34)	0.98-19.09 (41)	0.96-18.74 (75)
33	0.47-3.15 (27)	0.27-3.80 (28)	0.25-3.63 (55)	0.99-15.90 (27)	0.89-16.87 (28)	0.91-16.31 (55)
34	0.62-3.69 (28)	0.78-5.56 (19)	0.74-4.65 (47)	0.94-17.66 (28)	1.13-17.59 (19)	0.96-17.80 (47)
35	0.64-3.97 (22)	0.52-3.61 (20)	0.54-3.89 (42)	1.02-18.12 (22)	9.77-17.95 (20)	1.25-18.51 (42)
36	0.62-4.11 (38)	0.27-5.13 (25)	0.49-4.55 (63)	0.85-16.23 (38)	1.23-20.17 (25)	0.90-17.15 (63)
37	0.77-6.29 (22)	0.32-4.24 (25)	0.55-4.26 (47)	9.31-15.52 (22)	0.97-16.86 (25)	1.03-16.69 (47)
38	1.11-4.37 (17)	0.51-3.32 (16)	0.78-4.02 (33)	0.98-16.13 (17)	0.91-17.26 (16)	0.89-17.24 (33)
39	0.07-4.61 (11)	0.71-3.13 (9)	0.13-4.42 (20)	3.83-23.67 (11)	10.14-16.33 (9)	5.36-22.67 (20)
40	1.62-2.50 (8)	1.08-3.42 (7)	1.09-3.23 (15)	3.12-15.34 (8)	2.54-18.13 (7)	0.96-17.68 (15)

*GA, gestation age.

### Fetal sex differences in maternal thyroid function parameters

The results of the univariate analyses were shown in the **Supplementary Material STables 2**, **3**. **Table 3** demonstrated the sex-specific TSH and FT4 values in different gestational weeks using the non-adjusted and multivariate adjusted models. Compared with males, maternal serum TSH was consistently lower in pregnancies with females, both before and after model adjustment. After adjustment for confounders, the median TSH level was 0.11 mIU/L lower (*P*=0.00), and FT4 was 0.14 pmol/L higher (*P*=0.00) for mothers with female fetuses than those with males during gestational weeks 9-12.

**Table 3:** Effect of fetal sex on maternal thyroid hormone in different gestational weeks.

GA (week)		TSH (Median)				FT4 (Median)			
		Crude model		Adjusted model*		Crude model		Adjusted model*	
		Estimate	*P*	Estimate	*P*	Estimate	*P*	Estimate	*P*
1-8	Male	REF ^#^		REF		REF		REF	
	Female	-0.03	0.04	-0.01	0.59	0.06	0.17	0.08	0.11
9-12	Male	REF		REF		REF		REF	
	Female	-0.11	0.00	-0.11	0.00	0.11	0.00	0.14	0.00
13-28	male	REF		REF		REF		REF	
	female	-0.01	0.29	-0.02	0.05	-0.14	0.07	-0.08	0.21
29-40	male	REF		REF		REF		REF	
	female	-0.10	0.35	-0.10	0.38	-0.21	0.45	-0.38	0.29

*Adjusted for maternal age, pre-pregnancy BMI, pregnancy stage, ethnicity, season, and time interval.

REF, Reference.

## Discussion

This study demonstrated that compared to pregnancies with male fetuses, the TSH upper limit reference range of women bearing female fetuses decreased more while their FT4 upper limit reference range increased more during gestational weeks 7 to 12. The median level of TSH was 0.11mIU/L lower in pregnancies with females compared with males during gestational weeks 9 to 12, both before and after model adjustment, while the median level of FT4 in female pregnancies was 0.14 pmol/L higher than male pregnancies after model adjustment.

There is increasing evidence that fetal sex may alter the metabolic milieu during pregnancy (19, 20). However, only a few studies have reported an association between fetal sex and maternal thyroid function parameters during pregnancy. Previously, Sitoris et al. (14) reported that in women without thyroid autoimmunity (TAI) during gestational weeks 9 to 13 median (IQR range) serum TSH in the FF (female fetus) group was lower than that in the MF (male fetus) group: 1.13 (0.72–1.74) vs 1.24 (0.71–1.98) mIU/L; *P* = 0.021. Our research results were indeed the same, indicating that female fetus pregnancies produced lower serum level of TSH during gestational weeks 9 to 12 than male fetus pregnancies. In addition, we also found the level of serum FT4 was higher in female fetus pregnancies during the same gestational period.

Nonetheless, the mechanisms underpinning the observed effects of fetal sex differences in maternal thyroid function parameters during pregnancy are unclear. Previous studies have speculated that this may be due to differences in hCG concentrations between mothers with male and female fetus. TSH and hCG both belong to glycoprotein hormones, which share the identical α subunits, and there are small differences in their β subunits, which allows hCG to affect thyroid cells in a largely similar manner to TSH (21). In the first trimester, the peak hCG levels in normal pregnancy, lead to a modest reduction in secretion of TSH (22, 23), which bear a mirror image to the hCG peak.

Fetal sex has been shown to have a significant influence on maternal serum hCG levels. As reported in several papers, higher hCG levels were found in women pregnant with a female fetus (24, 25). Yaron et al. (26) showed hCG levels were significantly higher (18.5%) in week 3 post-fertilization in the presence of a female fetus (*P* < 0.0002). Illescas et al. (27) found that free β-hCG values were significantly higher (*P* < 0.01) in pregnancies with female fetuses in the first trimester of pregnancy. While Gol et al. (25) showed hCG levels were significantly higher in pregnancies bearing female fetuses than those bearing male fetuses in the third trimester of pregnancy.

Concerning the results in the univariate analyses, maternal age, pre-pregnancy BMI, pregnancy stage, ethnicity, season, and time interval were associated with thyroid parameters. Previously, it has been reported that thyroid hormones are influenced by maternal age and seasonal fluctuations. Consistent with our research findings, the results of a meta-analysis showed that TSH seasonal dynamics differences were pronounced increases in TSH levels during winter in women. In the context of sampling time, TSH levels from early in the morning were significantly higher (*P* < 0.001) (28). In contrast, Zhang, et al. (29) and Wang, et al. (30) recently found that median TSH concentrations decreased from 7:00 in the morning to 12:00 at noon and 1:00 pm from infertile women and the female outpatient patients, respectively. These data add to the current conflicting evidence. Further studies will be needed to clarify the influencing factors of thyroid hormone levels in pregnant women.

From **Table 2** and **Figures 2**, **3**, in addition to the sexual dimorphism in maternal thyroid function parameters, we also found the TSH upper limit of the reference ranges had fluctuations during T2 and T3, while the FT4 levels were generally less affected. It has been reported that in general subjects aged 0-104 years old, the TSH reference interval varies significantly by age, sex, hour of day, and ethnicity, while the FT4 reference interval was not affected by age, sex, hour of day and time of year (18), indicating that FT4 are more stable than TSH levels, which was consistent with our results. However, the high level of TSH with a stable FT4 during T2 and T3 might also indicate the possibility of subclinical hypothyroidism in pregnant women during this time. This finding could provide clues on the timing of testing the thyroid function during the pregnancy.

There were two major strengths of our study. The first one was the large sample size. Second, the study was carried out in a single center, and all the hormone assays were performed in the same laboratory with standardized instruments and methods during the whole study period, which reduced bias in laboratory test results. There were also some limitations in this study. One of the limitations was that the observational nature of real-world data entails inherent limitations and pitfalls, such as confounding, bias, and chance, which needed to be dealt with. At the same time, the data at various time points were not self-sequential longitudinal data from each case throughout the gestation. Meanwhile, as a retrospective study, the sample collection time for hCG and thyroid hormone levels was inconsistent. As such, we were unable to measure hCG levels at the same timepoints as used for measuring thyroid function. Another limitation of the present study is that since the announcement of the China’s universal two-child policy in October 2015 and the implementation on January 1, 2016 (31, 32), some of the recruited women in this study were pregnant for their second time, which may result in some selection bias.

## Conclusions

We identified sexual dimorphism in maternal thyroid function parameters, especially during 9-12 weeks of pregnancy. Based on previous research, we speculated that it may be related to the higher HCG levels of mothers who were pregnant with girls during this period. However, it is only a hypothesis. Longitudinal studies are needed to determine if fetal sex-specific affects the maternal thyroid function across pregnancy. Obstetricians need to be aware of thyroid function changes and related impact factors during pregnancy.

## Data Availability

The raw data supporting the conclusions of this article will be made available by the authors, without undue reservation.
