# Supplementary material for: Fetal sexual dimorphism of maternal thyroid function parameters during pregnancy, a single center retrospective real-world study
Source: Front Endocrinol (Lausanne). 2024 Aug 16;15:1431621. doi: 10.3389/fendo.2024.1431621 (PMC11365044; doi:10.3389/fendo.2024.1431621)
Supplement: Supplementary file 1 [file Table1.pdf]

Supplementary Material

Fetal Sexual Dimorphism of Maternal Thyroid Function Parameters During Pregnancy, A Single Center Retrospective Real-world Study

Meiqin Wu [1#], Chunping Hu [2, 3#], Dan Huang [1, 4], Hao Ying [5*], Jing Hua [1*]

1 Shanghai Key Laboratory of Maternal Fetal Medicine, Department of Women's and Children's Health Care, Shanghai First Maternity and Infant Hospital, School of Medicine, Tongji University, Shanghai 201204, China

2 Department of Spine Surgery, Honghui Hospital, Xi'an Jiaotong University, Xi'an 710054, Shaanxi, China

3 Shaanxi Key Laboratory of Spine Bionic Treatment, Xi'an 710054, Shaanxi, China

4 Medical College, Soochow University, Suzhou, 215123, Jiangsu, China

5 Department of Obstetrics, Shanghai First Maternity and Infant Hospital, School of Medicine, Tongji University, Shanghai 201204, China

#These authors contributed equally to this work and share first authorship.

*Corresponding authors:

STable 1 Gender-specific sample size at each gestational week

GA (week)	Male (N, %)	Female (N, %)
1	9 (0.6)	6 (0.4)
2	1 (0.25)	3 (0.75)
3	11 (0.73)	4 (0.27)
4	182 (0.52)	169 (0.48)
5	711 (0.49)	727 (0.51)
6	1381 (0.51)	1344 (0.49)
7	3161 (0.53)	2852 (0.47)
8	3586 (0.53)	3225 (0.47)
9	3512 (0.53)	3156 (0.47)
10	2944 (0.52)	2728 (0.48)
11	2530 (0.53)	2283 (0.47)
12	3122 (0.53)	2810 (0.47)
13	2720 (0.52)	2487 (0.48)
14	2269 (0.52)	2090 (0.48)
15	2648 (0.53)	2334 (0.47)
16	2271 (0.51)	2192 (0.49)
17	1741 (0.52)	1581 (0.48)
18	1370 (0.51)	1326 (0.49)
19	642 (0.53)	563 (0.47)
20	282 (0.54)	239 (0.46)
21	167 (0.49)	175 (0.51)
22	142 (0.54)	119 (0.46)
23	105 (0.54)	88 (0.46)
24	99 (0.66)	51 (0.34)
25	72 (0.58)	52 (0.42)
26	81 (0.59)	56 (0.41)
27	60 (0.54)	52 (0.46)
28	55 (0.58)	40 (0.42)
29	36 (0.54)	31 (0.46)
30	38 (0.5)	38 (0.5)
31	42 (0.55)	35 (0.45)
32	34 (0.45)	41 (0.55)
33	27 (0.49)	28 (0.51)
34	28 (0.6)	19 (0.4)
35	22 (0.52)	20 (0.48)
36	38 (0.6)	25 (0.4)
37	22 (0.47)	25 (0.53)
38	17 (0.52)	16 (0.48)
39	11 (0.55)	9 (0.45)
40	8 (0.53)	7 (0.47)

GA: gestation age

STable 2 Univariate analysis for TSH

Variables	P2.5		P25		P50		P75		P97.5	
	Estimate	*P*	Estimate	*P*	Estimate	*P*	Estimate	*P*	Estimate	*P*
**Age**	0.00	0.09	-0.01	0.00	-0.01	0.00	-0.01	0.00	-0.01	0.06
**GA (week)**	0.00	0.18	0.02	0.00	0.02	0.00	0.02	0.00	0.04	0.00
**BMI**	0.01	0.00	0.03	0.00	0.02	0.00	0.01	0.00	0.02	0.02
**Pregnancy stage**										
First trimester	REF		REF		REF		REF		REF	
Second trimester	0.00	0.74	0.25	0.00	0.28	0.00	0.30	0.00	0.47	0.00
Third trimester	0.28	0.00	0.57	0.00	0.55	0.00	0.66	0.00	0.81	0.00
**Race**										
Han	REF		REF		REF		REF		REF	
Hui	0.05	0.21	0.01	0.92	0.01	0.92	0.08	0.37	0.10	0.79
Man	-0.01	0.84	-0.03	0.73	0.09	0.19	0.17	0.24	0.55	0.02
Menggu	-0.02	0.75	0.05	0.47	0.00	0.98	0.01	0.97	0.21	0.60
Miao	0.04	0.71	0.12	0.38	0.28	0.02	0.60	0.08	0.58	0.00
Zhuang	0.07	0.52	0.30	0.00	0.34	0.00	0.53	0.00	0.00	0.99
Tujia	-0.02	0.63	-0.17	0.20	-0.27	0.00	-0.32	0.00	-0.76	0.01
Other	0.03	0.51	0.00	0.95	-0.03	0.66	0.03	0.66	-0.21	0.37
**Season**										
Summer	REF		REF		REF		REF		REF	
Spring	0.00	0.58	0.01	0.30	0.02	0.07	-0.01	0.64	-0.01	0.83
Autumn	0.00	0.67	-0.01	0.40	0.00	0.93	-0.01	0.64	0.02	0.71
Winter	0.00	0.49	0.01	0.65	0.03	0.01	0.02	0.17	0.04	0.47
**Time interval**										
Ante meridiem	REF		REF		REF		REF		REF	
Post meridiem	-0.01	0.41	0.03	0.08	0.07	0.00	0.12	0.00	0.12	0.19
Nighttime	-0.03	0.62	-0.35	0.11	0.47	0.06	0.37	0.02	-0.06	0.71

GA: gestation age; BMI: body mass index; REE: reference

STable 3 Univariate analysis for FT4

Variables	P2.5		P25		P50		P75		P97.5	
	Estimate	*P*	Estimate	*P*	Estimate	*P*	Estimate	*P*	Estimate	*P*
**Age**	0.00	0.00	0.02	0.00	-0.02	0.00	-0.03	0.00	-0.01	0.30
**GA (week)**	-0.02	0.00	-1.30	0.00	-0.29	0.00	-0.25	0.00	-0.25	0.00
**BMI**	-0.01	0.00	-0.16	0.00	-0.13	0.00	-0.14	0.00	-0.21	0.00
**Pregnancy stage**										
First trimester	REF		REF		REF		REF		REF	
Second trimester	-0.22	0.00	-13.93	0.00	-3.55	0.00	-2.71	0.00	-3.07	0.00
Third trimester	-0.29	0.00	-4.09	0.00	-3.33	0.00	-3.60	0.00	-4.47	0.00
**Race**										
Han	REF		REF		REF		REF		REF	
Hui	-0.01	0.80	-1.82	0.20	-0.27	0.29	-0.27	0.23	-0.98	0.02
Man	-0.05	0.27	-0.69	0.47	-0.29	0.14	-0.37	0.07	-0.11	0.94
Menggu	0.05	0.49	-0.39	0.66	-0.67	0.01	-0.99	0.01	-1.15	0.03
Miao	-0.04	0.70	-0.01	0.99	-0.43	0.24	-0.66	0.01	0.19	0.86
Zhuang	-0.12	0.07	-0.83	0.65	-0.27	0.23	-0.55	0.10	1.82	0.04
Tuajia	-0.03	0.63	-12.14	0.00	-0.56	0.33	0.22	0.73	0.98	0.17
Other	-0.02	0.68	0.22	0.53	-0.18	0.28	-0.19	0.41	-1.04	0.00
**Season**										
Summer	REF		REF		REF		REF		REF	
Spring	-0.01	0.29	-1.00	0.00	0.03	0.42	0.22	0.00	0.23	0.06
Autumn	-0.01	0.32	-0.32	0.00	0.16	0.00	0.22	0.00	0.30	0.02
Winter	0.01	0.30	-1.30	0.00	-0.23	0.00	-0.11	0.01	-0.07	0.58
**Time interval**										
Ante meridiem	REF		REF		REF		REF		REF	
Post meridiem	0.04	0.05	0.55	0.00	0.20	0.00	0.33	0.00	0.91	0.00
Nighttime	0.02	0.95	-0.01	0.99	-0.99	0.15	1.97	0.04	18.04	0.00

GA: gestation age; REE： Reference
